# Cardiac self-limiting rhabdomyomas in a neonatal patient with tuberous sclerosis complex: a case report with negative genetic testing

**DOI:** 10.3389/fped.2023.1263631

**Published:** 2023-10-10

**Authors:** Huatao Zhou, Zilong Zheng, Zhi Tu, Yichen Li, Jinfu Yang, Chengming Fan

**Affiliations:** Department of the Cardiovascular Surgery, The Second Xiangya Hospital, Central South University, Changsha, China

**Keywords:** tuberous sclerosis complex (TSC), cardiac tumor, rhabdomyomas, subependymal nodules, mTOR inhibitor

## Abstract

**Background:**

Tuberous Sclerosis Complex (TSC) is a hereditary condition that leads to the development of non-malignant neoplasms in various organs, including cardiac rhabdomyomas, which can cause significant complications.

**Case presentation:**

This report describes the case of a 15-day-old male neonate who was hospitalized due to intracardiac masses and brain lesions, despite the absence of TSC gene mutations. The patient's mother exhibited facial angiofibromas, a common feature of TSC. Over a 2-year follow-up period, spontaneous regression of the cardiac tumor was observed.

**Conclusions:**

This case illustrates that not all TSC cases exhibit detectable TSC gene mutations. Current treatment strategies, such as mTOR inhibitors, offer potential effectiveness in managing associated cardiac rhabdomyomas. Further research should focus on evaluating the therapeutic potential of these inhibitors.

## Background

Tuberous sclerosis complex (TSC) is a rare and complex genetic disorder caused by mutations in the TSC1 or TSC2 genes (1). These genetic changes result in continuous hyperactivation of the mechanistic target of rapamycin (mTOR) pathway, leading to uncontrolled cell growth and proliferation (2). TSC manifests as hamartomas, benign growths, in various organs, including the heart, where they are referred to as cardiac rhabdomyomas (3).

Cardiac rhabdomyomas are the most common primary cardiac tumors in children, accounting for 45% of cases (4, 5). There is a strong association between cardiac rhabdomyomas and TSC, with approximately 70%–90% of children with cardiac rhabdomyomas also being diagnosed with TSC. Conversely, around 90% of TSC patients under the age of two exhibit either single or multiple cardiac rhabdomyomas (4, 6).

Although rhabdomyomas are typically benign, they can lead to serious complications in some cases, including arrhythmias and obstruction of heart chambers, resulting in heart failure (7). While most of these tumors regress naturally within the first few years of life, surgical intervention may be necessary for life-threatening hemodynamic complications (8). However, congenital cardiac rhabdomyomas are of particular interest due to the spontaneous regression observed in more than half of the cases (9).

In addition to the heart, TSC has a significant impact on the central nervous system, causing various structural and functional abnormalities. Structural manifestations include cortical tubers and subependymal nodules, while functional implications encompass seizures, cognitive impairment, and behavioral alterations (10). These cerebral abnormalities are typically detected using magnetic resonance imaging (11).

In addition to the heart and brain, TSC affects the skin, kidneys, lungs, and eyes, resulting in a wide range of phenotypic manifestations of varying severity (12). Skin features, in particular, are a common major feature, occurring in 70% of TSC patients (13).

## Case presentation

**Figure 1:**
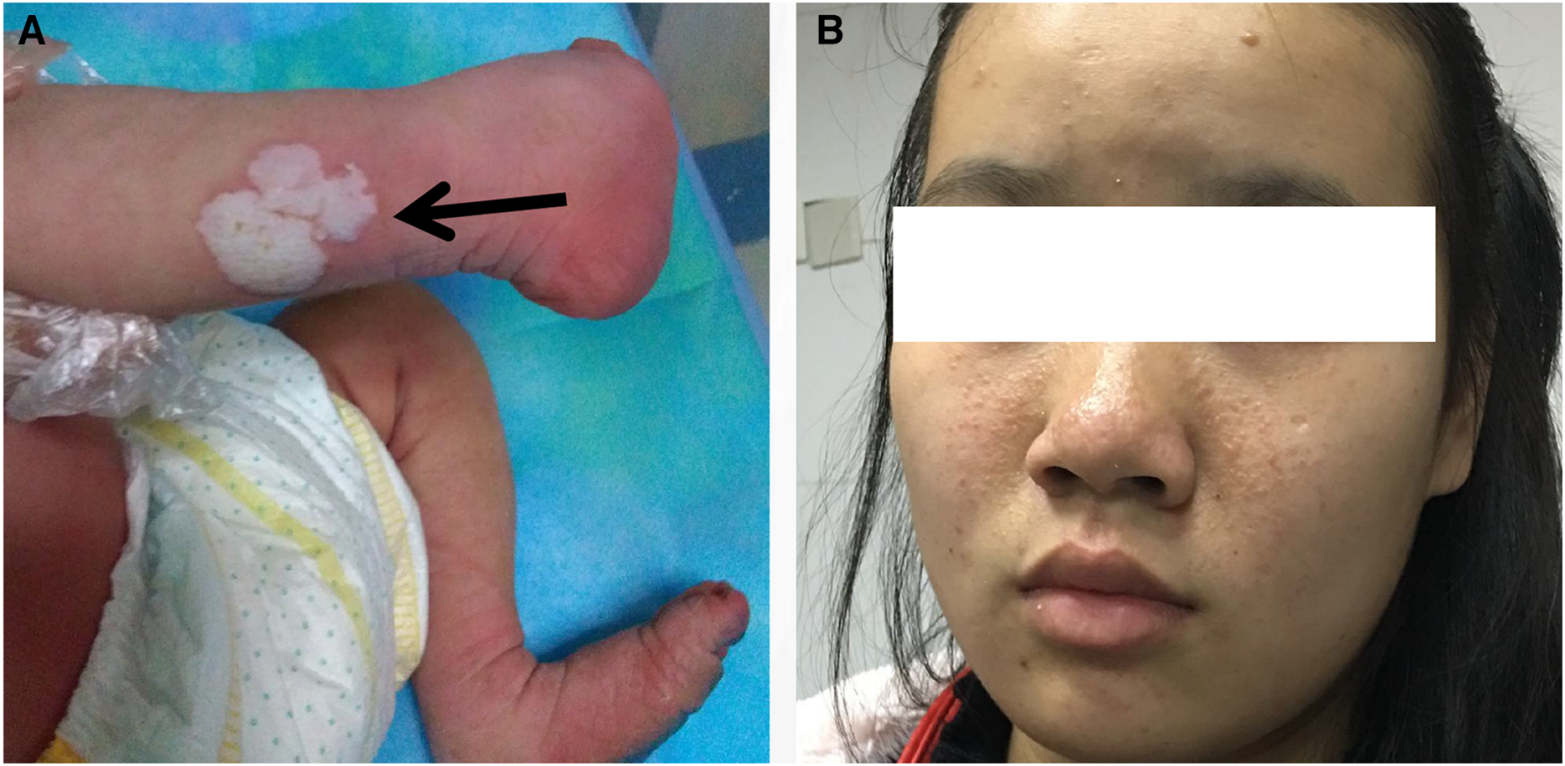
Images of the patient and the patient's mother. (**A**) On the posterior lateral side of the patient's right lower leg, a pale solid plaque measuring 2 × 2 cm in size was observed. (**B**) The patient's mother presented with facial angiofibromas on both malar prominences and in the nasolabial folds.

**Figure 2:**
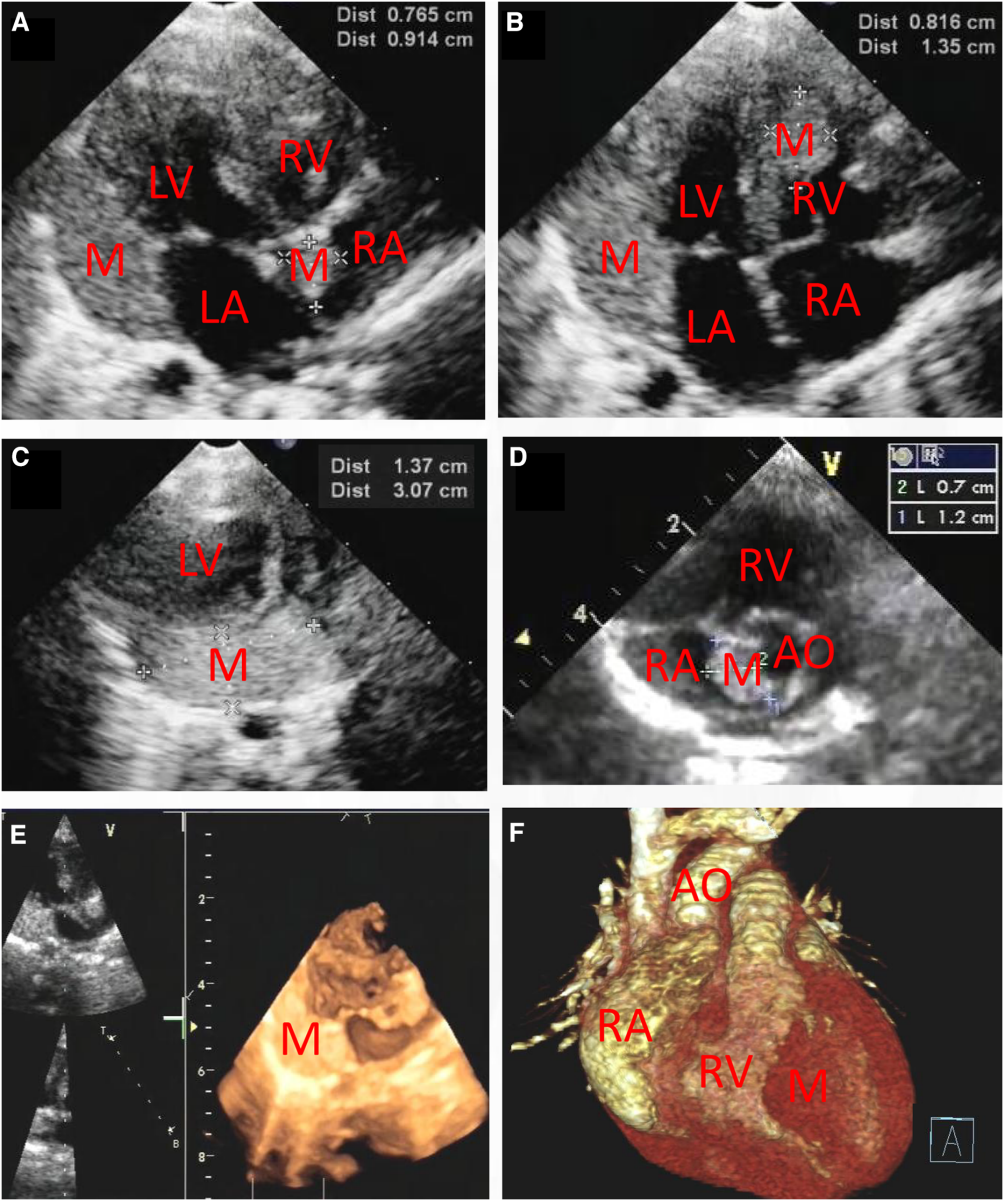
Transthoracic echocardiography (**A–D**) showing that there are multiple hyperechogenic masses within the atrial septum (0.765 cm × 0.914 cm and 0.7 cm × 1.2 cm), the interventricular septum (0.816 cm × 1.35 cm) and the inferolateral wall of the left ventricle (1.37 cm × 3.07 cm). Three-dimensional echocardiography (**E**) and Three-dimensional cardiac computed tomography angiography (**F**) confirmed the detection of cardiac multiple masses. M, mass; RA, right atrium; RV, right ventricle; LA, left atrium; LV, left ventricle; AO, ascending aorta.

**Figure 3:**
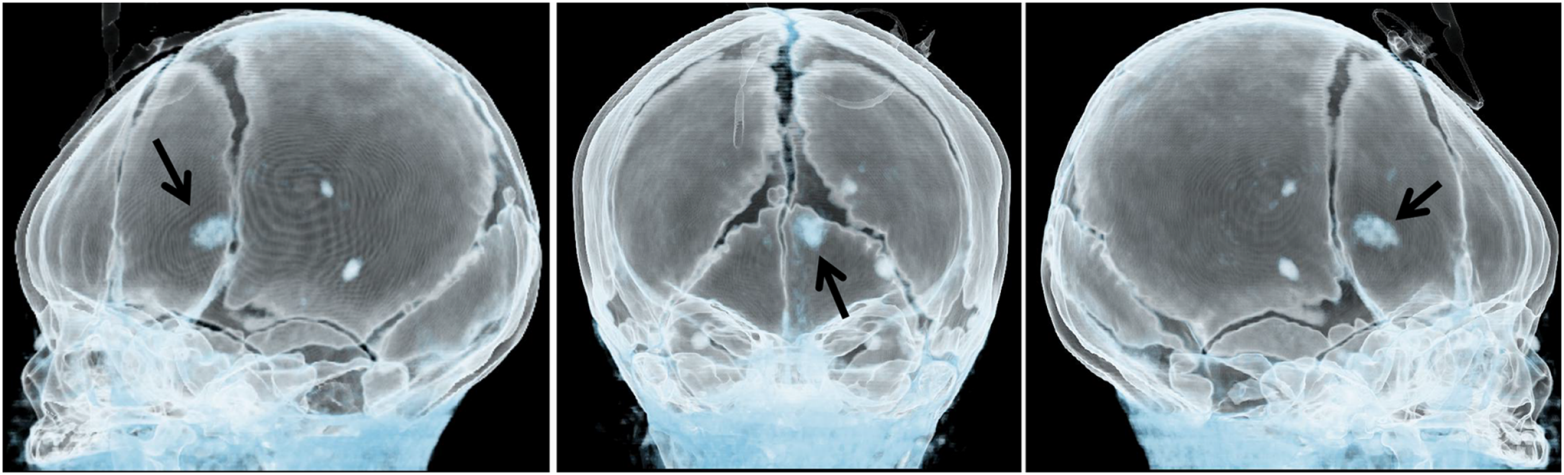
Three-dimensional cerebral computed tomography angiography showing multiple nodular high-density shadows with distinct borders. These shadows were distributed across several regions, including the left fronto-parietal lobe, right frontotemporal lobe, and bilateral subependyma of the lateral ventricles. A prominent lesion, measuring approximately 1.2 × 1.4 cm, was observed adjacent to the frontal horn of the right lateral ventricle (arrow).

This report presents the case of a 15-day-old male neonate with intracardiac masses. The neonate's initial medical history, including family history of cardiovascular disease, did not reveal any significant findings. On physical examination, a pale solid plaque measuring 2 × 2 cm was observed on the posterior lateral side of the neonate's right lower leg (Figure 1A). The patient's mother exhibited facial angiofibromas on both malar prominences and in the nasolabial folds (Figure 1B). Transthoracic echocardiography of the neonate confirmed the presence of multiple neoplasms within the interatrial septum, interventricular septum, right ventricular outflow tract, and left ventricle posterior wall (Figures 2A–D, see Supplementary Video S1). The largest nodule, measuring approximately 31 × 14 mm, was located in the inferolateral wall of the left ventricle, extending from the level of the mitral valve to the papillary muscle, with unclear borders and protrusion towards the pericardium (Figure 2C). Three-dimensional echocardiography (Figure 2E, see Supplementary Video S1) and Three-dimensional cardiac computed tomography angiography (Figure 2F, see Supplementary Video S2) further confirmed the presence of multiple tumorous masses in the heart. Three-dimensional cerebral computed tomography angiography revealed multiple nodular, high-density shadows with distinct borders in various regions, including the left fronto-parietal lobe, right frontotemporal lobe, and bilateral subependyma of the lateral ventricles. A prominent lesion measuring approximately 1.2 × 1.4 cm was observed adjacent to the frontal horn of the right lateral ventricle (Figure 3, see Supplementary Video S3). The 2018 World TSC Conference, hosted in Dallas, Texas, USA, introduced revisions to the clinical diagnostic criteria for TSC, as illustrated in Supplementary Table S1 (14). Based on the shagreen patche (Major Criteria 4), cardiac rhabdomyomas (Major Criteria 9) and multiple cortical tubers (Major Criteria 6), a confident diagnosis of TSC was established for this case, despite the absence of mutations in both the TSC1 and TSC2 genes (59 genes in total, with primers provided in Supplementary Table S2) of the neonate. As there was no significant influence on hemodynamics, close monitoring was recommended instead of open-heart surgery. During the 2-year follow-up period, the cardiac masses remained undetected, and the patient remained asymptomatic. No intellectual disability was detected.

## Discussion

Primary pediatric cardiac tumors are predominantly benign, with rhabdomyoma, fibroma, and teratoma being the most common types (15, 16). The association between rhabdomyoma and TSC, an autosomal dominant neuroectodermal disorder, is particularly strong, affecting approximately 1 in 5,000–10,000 live births (1). TSC and related rhabdomyomas result from mutations in the tumor suppressor genes TSC1 or TSC2, leading to a deficiency of hamartin or tuberin proteins (17). The loss of these proteins dysregulates the mTOR pathway, promoting uncontrolled cell proliferation and contributing to the development of hamartomatous growth in multiple organs (2).

Cardiac rhabdomyomas are generally benign and tend to regress naturally (4). The regression of these tumors, particularly in younger patients and smaller-sized tumors, is attributed to the withdrawal of maternal estrogen post-birth (18). The decline in maternal hormone support leads to the degradation of myofilaments, cytoplasmic glycogen vacuolization, and apoptosis, resulting in tumor regression (18). Therefore, asymptomatic rhabdomyomas often require only close surveillance (19). Clinical intervention is only necessary in cases of hemodynamic impairment and intractable arrhythmia (19).

Genetic testing for TSC is recommended for patients diagnosed with multiple cardiac rhabdomyomas or those exhibiting potential rhabdomyomas, as well as their family members, to evaluate familial or sporadic occurrence (20). However, genetic testing is not mandatory for TSC diagnosis, as approximately 10%–15% of clinically defined TSC cases lack detectable TSC1 or TSC2 mutations (20), as observed in this case report. De novo mutation other than TSC1 or TSC2 mutations may be the reason. Next-generation sequencing (NGS) is now preferred over previous DNA detection techniques due to its improved sensitivity and diagnostic yield (21). The patient's parent declined further diagnostic procedures, including a skin biopsy, as they believed that the diagnosis could be made through observation.

Regarding treatment options, mTOR inhibitors have shown promise in managing various tumor types associated with TSC, such as refractory epilepsy, renal angiomyolipomas, pulmonary lymphangioleiomyomatosis, and facial angiofibromas (22–25). Current research primarily focuses on targeted therapies that aim to inhibit specific pathways involved in tumorigenesis, including the epidermal growth factor receptor (EGFR), platelet-derived growth factor receptor (PDGFR), mTOR, and vascular endothelial growth factor (VEGF) pathways. These targeted agents have demonstrated promising evidence of disease activity (21). For example, imatinib, a tyrosine-kinase inhibitor, has shown potential in clinical trials and is being studied in combination with everolimus for potential synergistic effects (26).

The upcoming randomized trial (ORACLE) holds the promise of providing more robust and evidence-based outcomes regarding the effectiveness of mTOR inhibitors in the treatment of cardiac rhabdomyomas in children with TSC (27). This trial aims to provide valuable insights into the therapeutic potential of mTOR inhibitors and further advance our understanding of their role in managing cardiac rhabdomyomas associated with TSC (27).

## Conclusion

The presented case highlights that cardiac rhabdomyomas are part of TSC, and surgical removal is not always necessary. Close monitoring is the recommended approach for asymptomatic cardiac rhabdomyomas due to their characteristic of spontaneous regression.

## Data Availability

The datasets presented in this study can be found in online repositories. The names of the repository/repositories and accession number(s) can be found in the article/**Supplementary Material**.

## Supplementary material

Supplementary Video S1 Transthoracic echocardiography and three-dimensional echocardiography showing multiple cardiac masses.

Supplementary Video S2 Three-dimensional cardiac computed tomography angiography showing multiple cardiac masses.

Supplementary Video S3 Three-dimensional cerebral computed tomography angiography showing multiple nodular high-density shadows with distinct borders.
