# Supplementary material for: Cardiac self-limiting rhabdomyomas in a neonatal patient with tuberous sclerosis complex: a case report with negative genetic testing
Source: Front Pediatr. 2023 Oct 10;11:1263631. doi: 10.3389/fped.2023.1263631 (PMC10597626; doi:10.3389/fped.2023.1263631)
Supplement: Supplementary file 1 [file Table1.docx]

Supplementary table 1. Clinical Diagnostic Criteria of TSC

| Major Criteria | Minor Criteria |
| --- | --- |
| 1. Hypomelanotic macules (≥3; at least 5mm diameter) | 1. “Confetti” skin lesions |
| 2. Angiofibroma (≥3) or fibrous cephalicplaque | 2. Dental enamel pits (≥3) |
| 3. Ungual fibromas (≥2) | 3. Intraoral fibromas (≥2) |
| 4. Shagreen patch | 4. Retinal achromic patch |
| 5. Multiple retinal hamartomas | 5. Multiple renal cysts |
| 6. Multiple cortical tubers and/or radial migration lines* | 6. Nonrenal hamartomas |
| 7. Subependymal nodule (≥2) | 7. Sclerotic bone lesions |
| 8. Subependymal giant cell astrocytoma |  |
| 9. Cardiac rhabdomyoma |  |
| 10. Lymphangiomyomatosis (LAM)** |  |
| 11. Angiomyolipomas (≥2)** |  |

Definite TSC: 2 major features or 1 major feature with 2 minor features

Possible TSC: either 1 major feature or >2 minor features
* includes tubers and cerebral white matter radial migration lines

** a combination of the 2 Major clinical features LAM and angiomyolipomas without other

features does not meet criteria for a Definite Diagnosis
