# Supplementary material for: Cardiac self-limiting rhabdomyomas in a neonatal patient with tuberous sclerosis complex: a case report with negative genetic testing
Source: Front Pediatr. 2023 Oct 10;11:1263631. doi: 10.3389/fped.2023.1263631 (PMC10597626; doi:10.3389/fped.2023.1263631)
Supplement: Supplementary file 2 [file Table2.docx]

Supplementary table 2. Primers of the 59 genes used for TSC detection.

| Gene name | F（5'-3'） | R(5'-3') | bp |
| --- | --- | --- | --- |
| TSC1-3 | GAAACCCTCTTCATAAACTCG | GTGGCTCTAAAGTCAATCTCT | 455 |
| TSC1-4 | TGGATTTTGTGACAGGAAGC | TGGGAACAATGTCATCAGTG | 320 |
| TSC1-5 | TTTGTTTGCTAACGTGGTTT | TTCCTTGCTTTAAGTTGCCTA | 402 |
| TSC1-6 | TACTGTACAATGCCGATCCTG | ACGACTAATCCTTTTAAGCCAA | 442 |
| TSC1-7 | CTCCTCAATCTGTCTCCAAC | CCAGTCACAAAAGACCACAT | 439 |
| TSC1-8 | GCCCTTTATAATTTGTCAACCC | GCTAAATGTTATTAGTCCTCCG | 307 |
| TSC1-9 | GCTATCAGAGTTCCGTGGCT | CAATCAGGTAGCAGACCAAGG | 484 |
| TSC1-10 | TTGTCATCTGTATTGTGCTT | AAGATGAATCTAAGAGGCAAC | 567 |
| TSC1-11 | CTGTTGCCTCTTAGATTCAT | CAAGTTTACAACAGCAAGTG | 407 |
| TSC1-12 | CTGGGGCTTTCAGAATGTGT | GCAGAGGGATAGCAGACGAG | 334 |
| TSC1-13 | AACTACTTTACAATATGCTC | GGGGATACTACAAAAGACTG | 473 |
| TSC1-14 | GGCCTAAACATAATGAAACGA | CACCCATCTTGTTACCTCA | 581 |
| TSC1-15a | CACCCAAACTGCCTAGTCT | CTTCCAAAGCCCACTCTCG | 427 |
| TSC1-15b | AAAATTCCACCTCCGACGAGA | AGCCTAGAAGGACATCTGACA | 386 |
| TSC1-16 | AGGCTTCTCCTCTAACTCTC | AATGCTGACTTGGCAACACT | 378 |
| TSC1-17 | AGGGGGCTTGATTGAACCAT | AACTCTGACCTCCTCGGCTG | 328 |
| TSC1-18 | ACCTGCTCTTTAAGTTGTGA | GTGCTGCAGTTTATACCTGT | 606 |
| TSC1-19 | AAGAAAGTAGAGCCGTTGAG | TCTGAAGGAAGAATGTTAGC | 294 |
| TSC1-20 | CAGGTGTTCTGTGGGTGGTC | TGTCTGGGTCTGAAACGCTT | 450 |
| TSC1-21 | GCCTTCTCAGTCCTTCTTAC | AGATACAGACCAGCCAGAAT | 358 |
| TSC1-22 | CCATGTTCTCCAGCAGACTCA | GAATTGGCAGCTTAGTCCCA | 422 |
| TSC1-23a | GACTAGACAGGCTGCAACAC | CTGGCTCTCGCTCTTATTAC | 671 |
| TSC1-23b | CAGAGAAACCCCCACACCAG | AATGCCAGATCCAAAAACCGT | 528 |
| TSC2-2 | ATGTCCCCATTCCTGTTTCGT | TCCCACAGAACCTGGTGCAAG | 279 |
| TSC2-3 | ACATTCTCAACTCCTCGGGAT | AAGTGCAAACCAGATCATCGG | 694 |
| TSC2-4 | AGCCGTTCCCTGTACGAAGCC | GCCCATCAGCCCCTAGACCCT | 432 |
| TSC2-5 | CTCCCCATACGCCGCTCTG | CAGCACCACCTCCCAGTGACA | 660 |
| TSC2-6 | AGATCCTAGTGTCCGTGCGTA | ACTTTATTCACTGCGGAGCTG | 392 |
| TSC2-7 | CTGAACACCCAGCACCGAGA | CCCGGCTATCCTGACTTACATGG | 401 |
| TSC2-8 | TTCCCACATGCCCGCTTGCC | GCCACCCCAAGAATCAGACAACC | 387 |
| TSC2-9 | CCAGCCCCTGACACGCATT | GTCAAGCCAAGACCCCGAGCC | 408 |
| TSC2-10 | CCGTCTCTCTGGGGAACACTT | CCCCAGCTGCAAAGCAACT | 294 |
| TSC2-11 | AAGCAAGCAGCTCTGACCCT | CCCCGGCTCACCTACTGCAT | 427 |
| TSC2-12 | GCCTCTGGTGCCAAGTCCAT | CAGCCCAGACACAAAGGCACT | 569 |
| TSC2-13 | AGCAAACCAGCCTCTCGACC | CCTGCTCATCGGGCATCCTG | 290 |
| TSC2-14 | AGGTCCTCTCATGACGCCACT | CAACAGAGACAGCCCGCCCAC | 348 |
| TSC2-15 | CTCATTGGCCTCCCTTGTGC | ACAGCCATCCGGTCACTCG | 289 |
| TSC2-16 | GTTCTCACGGCTGCTGACTC | ACTCCAACACAACGCAGATG | 260 |
| TSC2-17 | GCCCTGCAGCACACACTCCC | GCAGCTTCCAGGAACCACACC | 509 |
| TSC2-18 | TCCTGGGCCTGCACGAGCTT | TCCCCGCAGCAGGAACGGAAC | 294 |
| TSC2-19 | AGAGCCAAGTCTGTTCCGTTCCT | CTCCCTGTCCTCCTGGCCCAAC | 272 |
| TSC2-20 | CAGAGCCTCAGATGCTAGCTT | AAGGCACAATCTGCGCTCCA | 351 |
| TSC2-21 | CTCTGGCTACCCCGTGACCTG | CCCAAAGAGAGCTAGAAGCACCA | 393 |
| TSC2-22 | CGTGTCGGAATGCAACTGACC | GCACAGGTTACACAGCCCGAAC | 451 |
| TSC2-2324 | GGCTCCCCTGACCACCCTCT | CCCCAAACACCCTCCCACTG | 523 |
| TSC2-25 | AGGCTGTGTCTCTCGGTAGG | CACAGGACCCATTTCCACTC | 504 |
| TSC2-26 | CTGTCTCTTCCCCGCTAACT | AACCAGCCCGTGCTCATAC | 501 |
| TSC2-2728 | GGGTCTTTCCGAGCGAGGTC | GTCCCCAGGCTGGTACGAGG | 607 |
| TSC2-29 | ACGTGGCACCCTCGTACCAG | GAAGGCTCACCCCAGAGTCAG | 322 |
| TSC2-30 | GCATCAGGTAAGTGGTGGTC | GGGTGACTGGCAGAAAGATG | 304 |
| TSC2-31 | CAGAGATGGGTAAGGGGAGGT | AGGAGCCACATTGCCGTCAC | 338 |
| TSC2-32 | CACTCGGCACCGTGCTTCT | GTGGGCTTCCCCCTAAACAG | 684 |
| TSC2-33 | GTGGATGGCAGCAGTAAGCAG | CTAAGGAGCCCCCCGAGGT | 317 |
| TSC2-34 | GGGATGGAGGACAGATAGG | AGCCCACAGGGAGGAACAC | 768 |
| TSC2-3536 | GAACCTGGTGCCTCACTTGC | CCACGCTAACCTGTCACTCG | 760 |
| TSC2-37 | GCTGCTGGAATGGATGGTCTT | CCAGTGGTCCTCGGCTCTC | 548 |
| TSC2-38 | CTCCCATCCAGTCCTGCTAC | GCCAGTTACTCCTGACAGACAC | 668 |
| TSC2-394041 | CAACCAGGCAGTAGCCGAGA | GCTGAGGGAGCCCCATATTC | 562 |
| TSC2-42 | CAGACTTACTGCCCAAGCCGCCT | CCCCGCACCAAGCAGACAAA | 342 |
